# Social control is associated with increased reproductive skew in a wild mammal

**DOI:** 10.1098/rsbl.2024.0003

**Published:** 2024-06-05

**Authors:** Adriana A. Maldonado-Chaparro, Conner S. Philson, Xinping Zhang, Daniel T. Blumstein

**Affiliations:** ^1^ Department of Ecology and Evolutionary Biology, University of California, Los Angeles, CA 90095-1606, USA; ^2^ Department of Biology, Universidad del Rosario, Bogota D.C. 111221, Colombia; ^3^ Department of Migration, Max Planck Institute of Animal Behavior, Radolfzell 78457, Germany; ^4^ Rocky Mountain Biological Laboratory, Crested Butte, CO 81224, USA; ^5^ Centre for Research in Animal Behaviour, University of Exeter, Exeter EX4 4QG, UK

**Keywords:** despotism, egalitarianism, global reaching centrality, yellow-bellied marmot

## Abstract

In group-living species, reproductive variation among individuals of the same sex is widespread. By identifying the mechanisms underlying this reproductive skew, we gain fundamental insights into the evolution and maintenance of sociality. A common mechanism, social control, is typically studied by quantifying dominance, which is one of many attributes of sociality that describes how individuals exert influence on others and is an incomprehensive measure of social control as it accounts only for direct relationships. Here, we use the global reaching centrality (GRC), which quantifies the degree of hierarchy in a social network by accounting for both direct and indirect social relationships. Using a wild, free-living population of adult female yellow-bellied marmots (*Marmota flaviventris*), we found a positive relationship between the reproductive skew index and GRC: more despotic social groups have higher reproductive skew. The GRC was stronger predictor for skew than traditional measures of social control (i.e. dominance). This allows deeper insights into the diverse ways individuals control other group members’ reproduction, a core component in the evolution of sociality. Future studies of skew across taxa may profit by using more comprehensive, network-based measures of social control.

## 1. Introduction

Variation in the distribution of reproduction among same-sex individuals is widely observed across social mammals and is an important factor in the evolution of sociality [1–3]. In group-living species, where reproductive skew or reproductive inequality is most prevalent in females, selection tends to favour females with more reproductive capabilities, either via larger body size or by exerting greater control over resources for themselves at the expense of subordinates [3–8]. Thus, reproductive skew can be low among females when there are more evenly distributed opportunities for reproduction in a group (e.g. African lion [9]) or it can be monopolized by one or a few females, creating a high reproductive skew (e.g. meerkats [4]). However, the factors likely to affect female reproductive skew remain unclear.

Formal reproductive skew models fall into two general categories: transactional and compromise [1], both of which rely on the assumption that dominant individuals can influence subordinate reproduction [3,5]. To understand how reproduction is partitioned among individuals of the same sex within a social group, it is essential to properly estimate social control, which is a mechanism by which the social group regulates individual behaviour according to some rule [10,11]. Social control, with the aim of influencing the reproduction of other members of the group, can be exercised through aggression, eviction and infanticide [6,12–14].

Traditionally, social control is quantified by determining individual dominance ranks and the resulting dominance hierarchy. Dominance rank generally influences reproduction, with high-ranking individuals often having enhanced access to resources and reproductive success [6,15,16], as seen in some macaques (*Macaca* spp. [17–19]) and spotted hyenas (*Crocuta crocuta* [20,21]).

However, linear dominance, which describes direct relationships, is not the only mechanism for social control, which can also be exerted indirectly in a social group [22,23]. Indirect relationships have important consequences for individual fitness in a variety of systems [24]. In the context of social control and reproductive skew, an individual may be able to dominate or influence their indirect social partners (i.e. those they do not interact with directly) through those they do interact with directly [24,25]. Factoring in both direct and indirect relationships may provide a more comprehensive view of the mechanisms of social control and reproductive skew and will advance our understanding of the implications of social structure for the evolution of animal sociality.

We used social network analysis to specifically ask whether the degree of both direct and indirect social control in a social group is correlated with the female reproductive skew of the group. We used an adapted version of the global reaching centrality (GRC), a global network measure of group social structure quantifying the degree of hierarchy, accounting for both direct and indirect relationships [26]. The GRC is the normalized value of the difference between the maximum and the average value of the reach centralities of the network [26]. As the GRC increases, the network becomes less egalitarian and more despotic. Although research in animal social networks primarily considers positive relationships between individuals, the use of antagonistic relationships (i.e. aggression) may be a more appropriate measure of social control in networks (e.g. [25]). Thus, measures of power and centrality, calculated from a social network measure like the GRC, can be used to identify the proximate determinants of reproductive skew.

If one or a few individuals have a disproportionate influence over others in the social group, based on how the group is connected, the GRC of the group increases, and the group is under greater despotic control. By contrast, if individuals are similarly connected to other members of the group, the GRC is lower, and the group can be described as being egalitarian. Assuming that highly central or dominant individuals can influence the reproduction of other members of their group [1,3,5], we predict a positive relationship between GRC and reproductive skew.

## 2. Methods

### (a) Study system

We explored the relationship between social control and female reproductive skew in a wild, free-living population of yellow-bellied marmots (*Marmota flaviventris*), a facultative social species [27–29] that live in matrilineal social groups (mother : daughter : sister groups) and exhibit a harem-polygynous mating system [30]. Social groups are composed of one or more adult males and females (>2 years old), yearlings (1 year old) and pups (<1 year old) [31]. Colonies are composed of one or multiple social groups that vary in composition and size [32]. Although groups are kin structured, adult females compete with other females for resources and reproductive success [30]. Females can reproduce at age 2 and their litters emerge above ground *ca* 25 days after birth in June and July [30]. Pups remain in their natal colony until the following summer after which most males leave, whereas about half of the females stay in their natal colony [30]. Marmots may have low levels of reproductive skew [33,34]. Female marmots are known to reproductively suppress younger individuals [35], although the mechanisms involved are not fully understood. In marmots, reproductive skew is not a result of individual differences in body mass or lack of reproductive capacity [34], but may result from reproductive suppression by older, more dominant females ([30], but see [36]). Thus, the GRC is a relevant global social network measure for this system. Further, other global social network measures of group social structure have been shown to relate to reproductive success in this system [37].

We studied a population in and around the Rocky Mountain Biological Laboratory, Gothic, CO, USA [38,39]. From 2003 to 2020, marmots were live trapped between mid-April and early September, under permits issued by the Colorado Division of Wildlife (TR917, renewed annually) with ethical approval from UCLA IACUC (2001-191-01, renewed annually). Each trapped individual was weighed and sexed, and we recorded its reproductive status, which, in females, was determined based on the nipple development [40]. Additionally, we took a hair sample from each new individual in our population for later genetic analysis and offspring assignment. Social observations aboveground were conducted from mid-April to early September, during hours of peak behavioural activity (from 07.00 to 10.00 in the morning and 16.00 to 19.00 in the afternoon). We used an all-occurrence sampling scheme [41] where, for each interaction, we recorded the time, type (i.e. affiliative or agonistic), the initiator and recipient, and the location. We considered agonistic interactions as negative interactions that included aggression and displacements (i.e. one individual moves away in response to the approach of another individual). It is noteworthy that agonistic interactions are relatively rare in marmots but are common among relatives who share space and engage in comparatively more affiliative interactions [41]. Moreover, agonistic interactions are often directed towards close kin [30].

### (b) Social control

Given we are interested in female–female social control and reproductive skew, and only adults are reproductive in this system, we used only adult female social interactions to construct social networks. Because marmots share space and burrows with a subset of individuals at each colony, social groups were based on space-use overlap (two individuals seen or trapped at the same location and time, or observed using the same burrow, within a 1 day interval). Using SOCPROG (v. 2.9 [42]), we determined annual simple-ratio pairwise association indices for adult females based on this space-use overlap [43]. We used these indices in the random walk community detection algorithm Map Equation to identify social group membership [44–47].

We constructed directed and weighted social networks based on antagonistic interactions between adult females recorded during the entire active season (from mid-April to early September) using ‘igraph’ (v. 1.2.11 [44]) in R (v. 4.2.0 [48]) for each Map Equation defined group in each year. These networks included only individuals trapped more than five times in a year (to eliminate transient individuals) and interactions with known recipients and initiators (as the direction of aggression is important for social control). The exclusion of undirected interactions or between unidentified individuals should not significantly influence social network measures [49]. The relatively low rate of unknown individuals in our observations [50], which occurred over the entire active season of these marmots, facilitates the reliability of our social network measure [49,51,52]. From these antagonistic networks, we calculated the GRC for groups of three or more adult females because GRC cannot be calculated for groups of two (electronic supplementary material, figure S1; code available on OSF [53]).

We also calculated a traditional measure of linear dominance hierarchies, Landau’s corrected index, *h′* [54,55], to compare with the GRC. The *h*′ index corrects for matrices that contain unknown relationships (e.g. when two individuals were not observed to interact) as well as when there are tied relationships, when individuals have an equal number of directed submissive behaviours. The *h*′ index is the mean value of the Landau’s index calculated for 10 000 permutations of the dominance matrix [55].

### (c) Reproductive skew

We quantified the reproductive output of each adult female (>1 year old) in a given year using a pedigree based on parentage assignment in the program Cervus (v. 3.0 [56]). DNA extraction, genotyping and parentage assignment methods are described in Olson & Blumstein [57] and Blumstein *et al*. [58]. Although there is no consensus about which skew index is the best [59], we used the *M* index because it best accounts for group size in the calculation [60]. We calculated the *M* index of breeding female groups (i.e. groups of at least three adult females) using ‘SkewCalc’ (v. 1.0 [60,61]) in R.

### (d) Statistical analysis

To examine the social correlates of female reproductive skew, we fitted a linear mixed model using the *M* index as the response variable. GRC, *h′* index and adult female social group size were included as fixed effects, with colony and year as random effects. We fitted three subsequent models, one with just GRC and group size as fixed effects, one with just *h′* index and group size, and one with just group size as a fixed effect (all three models also included colony and year as random effects). These three subsequent models were to better understand the relationship of the GRC’s with skew and assess model fit.

In all models, GRC, *h′* index, and group size were log-transformed and then standardized (mean-centred and divided by 1 s.d.). *M* index was also standardized. Because group size is associated with many social network measures [62], and group size had a correlation of 0.625 with GRC and 0.202 with *h′* in our dataset, we included group size as a fixed effect in all models. Model assumptions were checked, and the VIF for each fixed effect was less than 3.1. All models were fitted with ‘lme4’ (v. 1.1-33) in R. We report marginal and conditional *R* ^2^ values for each model, and the semi-partial marginal *R* ^2^ that estimates variance explained by each fixed effect were calculated using ‘partR2’ (v. 0.9.1 [63,64]). Using 100 parametric bootstrapping iterations, 95% confidence intervals for *R* ^2^ values were estimated.

## 3. Results

Between 2003 and 2020, we conducted 22 720 h of observation and recorded 3950 agonistic social interactions, 767 of which were between adult females and 372 of which were used to calculate the GRC and *hʹ* and were included in analysis (the number of interactions used decreased to 372 because we are exploring only adult female social groups with a minimum group size of 3). GRC and skew are measures of the group; thus, our level of analysis is the social group. Our final dataset contained 25 adult female social groups (that had GRC, *hʹ* and the *M* index calculated) across 11 years and three colony areas.

Marmot groups had a *hʹ* averaging 0.62 ± 0.28 (s.d.), confirming the tendency of adult females to live in organized linear hierarchies [54]. The mean adult female group size was 4.68 ± 2.58 (range = 3–11), and the mean number of females that weaned offspring in a group was 3.52 ± 1.96 (range = 1–7). The mean *M* index was 1.09 ± 0.93, indicating that reproduction in our female social groups was skewed [60].

**Figure 1.**
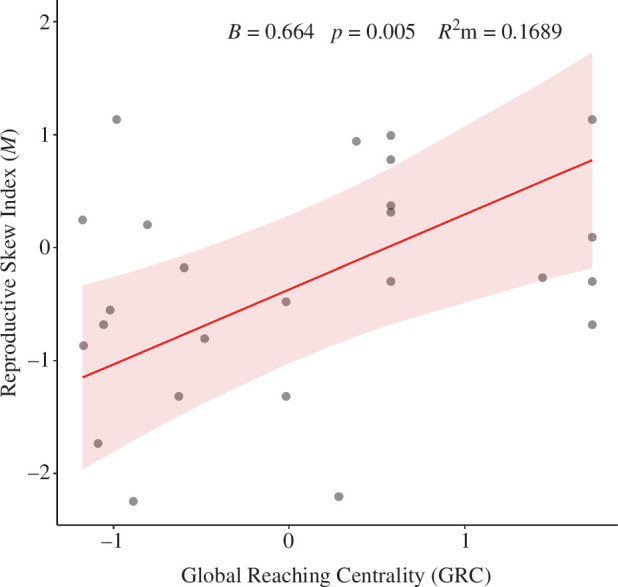
Relationship between social control, measured by the GRC index, and reproductive skew, measured by the *M* index (plotted as marginal effects with a 95% CI). The *M* index was scaled and the GRC was log transformed before being scaled. Generated with R package ‘sjPlot’ (v. 2.8.14 [65]).

In accordance with our *a priori* prediction, we found a statistically significant positive relationship between the GRC and the M index for reproductive skew (*B* = 0.664; *p* = 0.005; s.e. = 0.195; figure 1). Social group size also had a statistically significant positive relationship with reproductive skew (*B* = 0.688; *p* = 0.022; s.e. = 0.265). The more traditional measure of linear dominance, *h*ʹ, did not have a statistically significant association with skew (*B* = 0.253; *p* = 0.251; s.e. = 0.212; electronic supplementary material, figure S2). This model had a marginal *R* ^2^ value of 21.43% and a conditional *R* ^2^ value of 68.84%. GRC explained 16.89%, *h*ʹ 2.86%, and group size almost 0% of the marginal semi-partial *R* ^2^ variance (the latter of which should not be surprising given the *M* index already accounts for group size).

Subsequent models fitting only the GRC and group size, and only *h*ʹ and group size, reported the same relationships—a statistically significant positive relationship between the GRC and skew and no statistically significant relationship for *h*ʹ and skew (see electronic supplementary material, tables S1–S4). The model fitting only group size did not indicate a statistically significant relationship between skew and group size. The main model and the model for only GRC and group size were not statistically different in their fit (Akaike information criterion (AICc) = 69.24 and AICc = 68.931, respectively), whereas these two models were statistically a better fit than the model only fitting *hʹ* and group size (AICc = 77.43).

## 4. Discussion

We found support for reproduction within breeding groups being significantly more skewed as groups became more despotic (i.e. the GRC increased). This suggests that reproduction is controlled by one or a few individuals and that highly central individuals may prevent subordinates from breeding. We did not find support for a more traditional measure of dominance (or social rank), *hʹ*, being related to reproductive skew in this system (as was observed for individual adult female social rank and reproductive success in other systems: [6]). Previous work in this system shows that on an individual level, neither a female’s dominance rank [66] nor their position in their agonistic social network [67] were associated with their reproductive success. This suggests that within social groups, individual-level dominance and aggression as proximate mechanisms of social control are incomplete and that a more inclusive group-level measure incorporating direct and indirect interactions, such as the GRC, better quantifies social control and its role in reproductive skew.

Marmots within a social group maintained a strict dominance hierarchy, a pattern previously shown in this species [66] and in other matrilineal species such as macaques, *Macaca* spp. [17,18], and savannah baboons, *Papio cynocephalus* [68]. In hierarchical societies, dominance relationships may reflect individual differences in the female’s competitive ability to access resources that are related to the ability of individuals to gain reproductive benefits [69]. Marmots may share burrow systems [30] and foraging areas. However, while they are not restricted in their access to food, they may have to compete for access to high-quality burrows to successfully breed and survive winter [30].

Despite marmots maintaining a strict dominance hierarchy, linear dominance (i.e. social rank) did not explain statistically significant variance in adult female social group reproductive skew. Instead, a more inclusive measure of social control that accounts for both direct and indirect agonistic relationships, the GRC, did explain statistically significant variance. This is further seen in the effect sizes as the GRC explained 16.89% of the marginal variance alone, whereas social rank explained only 2.86% alone. Thus, the GRC better captures both the direct and indirect social mechanisms that dominant individuals may be using to control the reproductive behaviour of subordinates. Furthermore, our random effects of year and colony explained substantial variation (47.41%) in reproductive skew; this further suggests environmental and ecological features may play a role in mediating socially controlled reproductive skew [2].

Our results suggest that reproductive skew in yellow-bellied marmots may follow optimal skew models [3], as previously suggested by Allainé [33], which assume that a dominant individual has control over subordinate reproduction. Several mechanisms could be used by females to both directly and indirectly control the reproduction of their group mates. For instance, they could target aggressive behaviour to decrease subordinate mating success [1,3,69,70]. This could work by modifying endocrine levels to prevent pregnancies or to stimulate abortion [70,71]. In marmots, dominant females could socially suppress ovulation in subordinates, as has been described in marmosets (*Callithrix jacchus*) [72], engage in infanticidal behaviour [30] or monopolize access to key resources such as high-quality burrows, which affects the number of residents in a colony [30].

In conclusion, we have used social network analysis to better understand the proximate causes of reproductive skew in a wild, free-living mammal. We have shown that individuals may control the reproduction of other group members in ways that are consistent with expectations from optimal skew models. Our novel use of GRC as a measure to quantify socially mediated reproductive control shows that control emerges from more than simple dyadic aggressive interactions and requires knowledge of how individuals interact in the context of the entire group’s social structure. Moreover, the nature of our study system, where agonistic interactions are rare, expands the horizon for exploring conceptual and methodological frameworks that consider affiliative interactions to quantify social control. Thus, applying these techniques to other species will reveal the role of social structure in variation in reproductive success and the evolution of animal societies.

## Data Availability

Data and code to replicate analyses are available from OSF at [53]. Electronic supplementary material is available online at [73].

## References

[1] Johnstone RA . 2000 Models of reproductive skew: a review and synthesis (invited article). Ethology **106** , 5–26. (10.1046/j.1439-0310.2000.00529.x)

[2] Keller L , Reeve HK . 1994 Partitioning of reproduction in animal societies. Trends Ecol. Evol. **9** , 98–102. (10.1016/0169-5347(94)90204-6) 21236786

[3] Vehrencamp SL . 1983 A model for the evolution of despotic versus egalitarian societies. Anim. Behav. **31** , 667–682. (10.1016/S0003-3472(83)80222-X)

[4] Hodge SJ , Manica A , Flower TP , Clutton-Brock TH . 2008 Determinants of reproductive success in dominant female meerkats. J. Anim. Ecol. **77** , 92–102. (10.1111/j.1365-2656.2007.01318.x) 18031526

[5] Shen SF , Kern Reeve H . 2010 Reproductive skew theory unified: the general bordered tug-of-war model. J. Theor. Biol. **263** , 1–12. (10.1016/j.jtbi.2009.11.009) 19932705

[6] Shivani M , Huchard E , Lukas D . 2022 The effect of dominance rank on female reproductive success in social mammals. Peer Comm. Ecol. **2** , e48. (10.24072/pcjournal.158)

[7] Cant MA . 2000 Social control of reproduction in banded mongooses. Anim. Behav. **59** , 147–158. (10.1006/anbe.1999.1279) 10640376

[8] Clutton-Brock TH . 1998 Reproductive skew, concessions and limited control. Trends Ecol. Evol. **13** , 288–292. (10.1016/s0169-5347(98)01402-5) 21238306

[9] Packer C , Pusey AE , Eberly LE . 2001 Egalitarianism in female African lions. Science **293** , 690–693. (10.1126/science.1062320) 11474110

[10] Friedkin NE . 2004 Social cohesion. Annu. Rev. Sociol. **30** , 409–425. (10.1146/annurev.soc.30.012703.110625)

[11] Janowitz M . 1975 Sociological theory and social control. Am. J. Sociol. **81** , 82–108. (10.1086/226035)

[12] Bell MBV , Cant MA , Borgeaud C , Thavarajah N , Samson J , Clutton-Brock TH . 2014 Suppressing subordinate reproduction provides benefits to dominants in cooperative societies of meerkats. Nat. Commun. **5** , 4499. (10.1038/ncomms5499) 25047446 PMC4109011

[13] Clutton-Brock TH . 2021 Social evolution in mammals. Science **373** , eabc9699. (10.1126/science.abc9699) 34529471

[14] Larson SM , Ruiz-Lambides A , Platt ML , Brent LJN . 2018 Social network dynamics precede a mass eviction in group-living rhesus macaques. Anim. Behav. **136** , 185–193. (10.1016/j.anbehav.2017.08.019) 29887618 PMC5990275

[15] Hemelrijk CK . 1999 An individual-orientated model of the emergence of despotic and egalitarian societies. Proc. R. Soc. Lond. B **266** , 361–369. (10.1098/rspb.1999.0646)

[16] Leimar O , Bshary R . 2022 Effects of local versus global competition on reproductive skew and sex differences in social dominance behaviour. Proc. R. Soc. B **289** . (10.1098/rspb.2022.2081) PMC9709658 36448421

[17] Hemelrijk CK , Wantia J , Isler K . 2008 Female dominance over males in primates: self-organisation and sexual dimorphism. PLoS One **3** , e2678. (10.1371/journal.pone.0002678) 18628830 PMC2441829

[18] Silk JB , Samuels A , Rodman PS . 1981 Hierarchical organization of female Macaca radiata in captivity. Primates **22** , 84–95. (10.1007/BF02382559)

[19] Sukmak M , Wajjwalku W , Ostner J , Schülke O . 2014 Dominance rank, female reproductive synchrony, and male reproductive skew in wild Assamese macaques. Behav. Ecol. Sociobiol. **68** , 1097–1108. (10.1007/s00265-014-1721-z)

[20] Engh AL , Funk SM , Horn RCV , Scribner KT , Bruford MW , Libants S , Holekamp KE . 2002 Reproductive skew among males in a female-dominated mammalian society. Behav. Ecol **13** , 193–200. (10.1093/beheco/13.2.193)

[21] Holekamp KE , Smale L , Szykman M . 1996 Rank and reproduction in the female spotted hyaena. Reproduction **108** , 229–237. (10.1530/jrf.0.1080229) 9038781

[22] McDonald DB . 2007 Predicting fate from early connectivity in a social network. Proc. Natl Acad. Sci. USA **104** , 10910–10914. (10.1073/pnas.0701159104) 17576933 PMC1904119

[23] Ryder TB , Parker PG , Blake JG , Loiselle BA . 2009 It takes two to tango: reproductive skew and social correlates of male mating success in a lek-breeding bird. Proc. R. Soc. B **276** , 2377–2384. (10.1098/rspb.2009.0208) PMC2690469 19324732

[24] Brent LJN . 2015 Friends of friends: are indirect connections in social networks important to animal behaviour? Anim. Behav. **103** , 211–222. (10.1016/j.anbehav.2015.01.020) 25937639 PMC4415378

[25] McDonald DB , Shizuka D . 2013 Comparative transitive and temporal orderliness in dominance networks. Behav. Ecol. **24** , 511–520. (10.1093/beheco/ars192)

[26] Mones E , Vicsek L , Vicsek T . 2012 Hierarchy measure for complex networks. PLoS One **7** , e33799. (10.1371/journal.pone.0033799) 22470477 PMC3314676

[27] Blumstein DT , Armitage KB . 1999 Cooperative breeding in marmots. Oikos **84** , 369. (10.2307/3546418)

[28] Komdeur J , Ma L . 2021 Keeping up with environmental change: the importance of sociality. Ethology **127** , 790–807. (10.1111/eth.13200)

[29] Panaccio M , Ferrari C , Bassano B , Stanley CR , von Hardenberg A . 2021 Social network analysis of small social groups: application of a hurdle GLMM approach in the Alpine marmot (Marmota marmota) . Ethology **127** , 453–464. (10.1111/eth.13151)

[30] Armitage KB . 2014 Marmot biology. In Marmot biology: sociality, individual fitness, and population dynamics. Cambridge, UK: Cambridge University Press. (10.1017/CBO9781107284272)

[31] Downhower JF , Armitage KB . 1971 The Yellow-Bellied Marmot and the Evolution of Polygamy. Am. Nat. **105** , 355–370. (10.1086/282730)

[32] Maldonado-Chaparro AA , Hubbard L , Blumstein DT . 2015 Group size affects social relationships in yellow-bellied marmots (Marmota flaviventris). Behav. Ecol. **26** , 909–915. (10.1093/beheco/arv034)

[33] Allainé D . 2000 Sociality, mating system and reproductive skew in marmots: evidence and hypotheses. Behav. Processes **51** , 21–34. (10.1016/s0376-6357(00)00116-9) 11074309

[34] Armitage KB . 2003 Reproductive competition in female yellow-bellied marmots. In Adaptive strategies and diversity in marmots (eds R Ramousse , D Allainé ), pp. 133–142. Lyon, France: International Marmot Network.

[35] Armitage KB . 1998 Reproductive strategies of yellow-bellied marmots: energy conservation and differences between the sexes. J. Mammal. **79** , 385–393. (10.2307/1382969)

[36] Blumstein DT , Keeley KN , Smith JE . 2016 Fitness and hormonal correlates of social and ecological stressors of female yellow-bellied marmots. Anim. Behav. **112** , 1–11. (10.1016/j.anbehav.2015.11.002)

[37] Philson CS , Blumstein DT . 2023 Group social structure has limited impact on reproductive success in a wild mammal. Behav. Ecol. **34** , 89–98. (10.1093/beheco/arac102)

[38] Armitage KB . 2012 Sociality, individual fitness and population dynamics of yellow-bellied marmots. Mol. Ecol. **21** , 532–540. (10.1111/j.1365-294X.2011.05323.x) 22017671

[39] Blumstein DT . 2013 Yellow-bellied marmots: insights from an emergent view of sociality. Phil. Trans. R. Soc. B **368** , 20120349. (10.1098/rstb.2012.0349) 23569297 PMC3638452

[40] Armitage KB , Wynne-Edwards KE . 1997 Holarctic marmots as a factor of biodiversity. In Progesterone concentrations of wild-caught yellow-bellied marmots (eds KB Armitage , VY Rumiantsev ), pp. 41–47. Cheboksary, Russia: ABF Publishing House.

[41] Wey TW , Blumstein DT . 2010 Social cohesion in yellow-bellied marmots is established through age and kin structuring. Anim. Behav. **79** , 1343–1352. (10.1016/j.anbehav.2010.03.008)

[42] Whitehead H . 2009 SOCPROG programs: analysing animal social structures. Behav. Ecol. Sociobiol. **63** , 765–778. (10.1007/s00265-008-0697-y)

[43] Cairns SJ , Schwager SJ . 1987 A comparison of association indices. Anim. Behav. **35** , 1454–1469. (10.1016/S0003-3472(87)80018-0)

[44] Csardi G , Nepusz T . 2006 The Igraph software package for complex network research. In Interjournal, complex systems, 1695. See https://igraph.org.

[45] Pfau M , Degregori S , Johnson G , Tennenbaum SR , Barber PH , Philson CS , Blumstein DT . 2023 The social microbiome: gut microbiome diversity and abundance are negatively associated with sociality in a wild mammal. R. Soc. Open Sci. **10** , 231305. (10.1098/rsos.231305) 37830026 PMC10565414

[46] Rosvall M , Axelsson D , Bergstrom CT . 2009 The map equation. Eur. Phys. J. Spec. Top **178** , 13–23. (10.1140/epjst/e2010-01179-1)

[47] Rosvall M , Bergstrom CT . 2008 Maps of random walks on complex networks reveal community structure. Proc. Natl Acad. Sci. USA **105** , 1118–1123. (10.1073/pnas.0706851105) 18216267 PMC2234100

[48] R Development Core team . 2023 R: a language and environment for statistical computing, version 4.2.0. Vienna, Austria: R Foundation for Statistical Computing. See https://www.R-project.org/.

[49] Silk MJ , Jackson AL , Croft DP , Colhoun K , Bearhop S . 2015 The consequences of unidentifiable individuals for the analysis of an animal social network. Anim. Behav. **104** , 1–11. (10.1016/j.anbehav.2015.03.005)

[50] Philson CS , Blumstein DT . 2023 Emergent social structure is typically not associated with survival in a facultatively social mammal. Biol. Lett. **19** , 20220511. (10.1098/rsbl.2022.0511) 36918036 PMC10014246

[51] Davis GH , Crofoot MC , Farine DR . 2018 Estimating the robustness and uncertainty of animal social networks using different observational methods. Anim. Behav. **141** , 29–44. (10.1016/j.anbehav.2018.04.012)

[52] Sánchez-Tójar A , Schroeder J , Farine DR . 2018 A practical guide for inferring reliable dominance hierarchies and estimating their uncertainty. J. Anim. Ecol. **87** , 594–608. (10.1111/1365-2656.12776) 29083030

[53] Maldonado-Chaparro A , Philson CS , Zhang Z , Blumstein DT . 2023 Data for: Social control is associated with increased reproductive skew in a wild mammal. OSF (10.17605/OSF.IO/KF6YD) PMC11285479 38835239

[54] Fedigan L , Bergstrom M . 2010 Dominance among female white-faced capuchin monkeys (Cebus capucinus): hierarchical linearity, nepotism, strength and stability. Behaviour **147** , 899–931. (10.1163/000579510X497283)

[55] de Vries G . 1995 An improved test of linearity in dominance hierarchies containing unknown or tied relationships. Anim. Behav. **50** , 1375–1389. (10.1016/0003-3472(95)80053-0)

[56] Kalinowski ST , Taper ML , Marshall TC . 2007 Revising how the computer program CERVUS accommodates genotyping error increases success in paternity assignment. Mol. Ecol. **16** , 1099–1106. (10.1111/j.1365-294X.2007.03089.x) 17305863

[57] Olson LE , Blumstein DT . 2010 Applying the coalitionary-traits metric: sociality without cooperation in male yellow-bellied marmots. Behav. Ecol. **21** , 957–965. (10.1093/beheco/arq094)

[58] Blumstein DT , Lea AJ , Olson LE , Martin JGA . 2010 Heritability of anti‐predatory traits: vigilance and locomotor performance in marmots. J. Evol. Biol. **23** , 879–887. (10.1111/j.1420-9101.2010.01967.x) 20298440

[59] Nonacs P . 2003 Measuring the reliability of skew indices: is there one best index? Anim. Behav. **65** , 615–627. (10.1006/anbe.2003.2096)

[60] Ross CT , Jaeggi AV , Borgerhoff Mulder M , Smith JE , Smith EA , Gavrilets S , Hooper PL . 2020 The multinomial index: a robust measure of reproductive skew. Proc. R. Soc. B **287** , 20202025. (10.1098/rspb.2020.2025) PMC7657858 33023419

[61] Ross CT , Hooper PL . 2019 SkewCalc: estimation of reproductive skew. R package version 1.0. See https://github.com/Ctross/SkewCalc.

[62] Wasserman S , Faust K . 1994 Social network analysis: methods and applications. Cambridge, UK: Cambridge University Press. (10.1017/CBO9780511815478)

[63] Nakagawa S , Schielzeth H . 2013 A general and simple method for obtaining R2 from generalized linear mixed-effects models. Methods Ecol. Evol. **4** , 133–142.

[64] Stoffel MA , Nakagawa S , Schielzeth H . 2021 partR2: partitioning R2 in generalized linear mixed models. PeerJ **9** , e11414. 34113487 10.7717/peerj.11414 PMC8162244

[65] Lüdecke D . 2023 Package ‘sjPlot’. R package version, 2.8.14. See https://cran.r-project.org/web/packages/sjPlot/index.html.

[66] Huang B , Wey TW , Blumstein DT . 2011 Correlates and consequences of dominance in a social rodent. Ethology **117** , 573–585. (10.1111/j.1439-0310.2011.01909.x)

[67] Wey TW , Blumstein DT . 2012 Social attributes and associated performance measures in marmots: bigger male bullies and weakly affiliating females have higher annual reproductive success. Behav. Ecol. Sociobiol. **66** , 1075–1085. (10.1007/s00265-012-1358-8)

[68] Barton RA , Byrne RW , Whiten A . 1996 Ecology, feeding competition and social structure in baboons. Behav. Ecol. Sociobiol. **38** , 321–329. (10.1007/s002650050248)

[69] Reeve HK , Emlen ST , Keller L . 1998 Reproductive sharing in animal societies: reproductive incentives or incomplete control by dominant breeders? Behav. Ecol. **9** , 267–278. (10.1093/beheco/9.3.267)

[70] Creel S , Creel N , Wildt DE , Monfort SL . 1992 Behavioural and endocrine mechanisms of reproductive suppression in serenge dwarf mongooses. Anim. Behav. **43** , 231–245. (10.1016/S0003-3472(05)80219-2)

[71] Abbott DH . 1984 Behavioral and physiological suppression of fertility in subordinate marmoset monkeys. Am. J. Primatol. **6** , 169–186. (10.1002/ajp.1350060305) 31986838

[72] Saltzman W , Digby LJ , Abbott DH . 2009 Reproductive skew in female common marmosets: what can proximate mechanisms tell us about ultimate causes? Proc. R. Soc. B **276** , 389–399. (10.1098/rspb.2008.1374) PMC2592602 18945663

[73] Maldonado-Chaparro AA , Philson CS , Zhang X , Blumstein D . 2024 Supplementary material from: Social control is associated with increased reproductive skew in a wild mammal. FigShare (10.6084/m9.figshare.c.7227099) PMC11285479 38835239

